# Exosomal amyloid A and lymphatic vessel endothelial hyaluronic acid receptor-1 proteins are associated with disease activity in rheumatoid arthritis

**DOI:** 10.1186/s13075-017-1334-9

**Published:** 2017-05-31

**Authors:** Jihyung Yoo, Sang Kwang Lee, Mikyung Lim, Donghyuk Sheen, Eun-Hye Choi, Soon Ae Kim

**Affiliations:** 1 Department of Internal Medicine, School of Medicine, Eulji University, Daejeon, Korea; 2 Eulji Medi-Bio Research Institute, Eulji University, Daejeon, Korea; 3 Department of Pharmacology, School of Medicine, Eulji University, Daejeon, Korea

**Keywords:** Exosomes, LYVE-1, Rheumatoid arthritis, Amyloid A, Biomarker

## Abstract

**Background:**

Exosomes are thought to play an important role in exchanging information between cells. The proteins and lipids in exosomes play roles in mediating inflammatory and autoimmune diseases. The aim of this study was to identify exosomal candidate proteins that are related to other inflammatory parameters in rheumatoid arthritis (RA).

**Methods:**

The study population consisted of 60 patients with RA: 30 in the clinical remission (CR) group with a Disease Activity Score in 28 joints based on erythrocyte sedimentation rate (DAS28-ESR) ≤2.6 and 30 in the non-clinical remission (non-CR) group with a DAS28-ESR >2.6. Preparation of exosomes from patient serum samples was performed with the ExoQuick kit, and protein identification/quantification was performed using tandem mass tag labeling/mass spectrometry and an enzyme-linked immunosorbent assay. Comparisons between groups were made using Student’s *t* test or the Mann-Whitney *U* test, as appropriate. Spearman’s correlation coefficients (ρ) were calculated.

**Results:**

We identified six candidate proteins. Exosomal levels of amyloid A (AA) and lymphatic vessel endothelial hyaluronic acid receptor-1 (LYVE-1) differed between the CR and non-CR groups. Both serum and exosomal AA levels were higher in the non-CR group than in the CR group (*p* = 0.001). Significant positive correlations were found between exosomal AA and C-reactive protein (CRP) as well as between serum AA and CRP (ρ = 0.614, *p* = 0.001, and ρ = 0.624, *p* = 0.001, respectively). Although serum levels of LYVE-1 did not differ between the non-CR and CR groups, exosomal levels of LYVE-1 were lower in the non-CR group than in the CR group (*p* = 0.01). We identified positive correlations between serum/exosomal LYVE-1 and CRP only in the non-CR group (serum ρ = 0.376, *p* = 0.04; exosome ρ = 0.545, *p* = 0.002).

**Conclusions:**

Exosomal LYVE-1 shows potential for use as an additional marker of disease activity in patients with RA, and exosomes may carry other useful markers for RA.

## Background

Exosomes, membrane-bound vesicles 40–100 nm in diameter, are present in almost all biological fluids [1]. Exosomes are thought to play an important role in exchanging information between cells. Transmembrane exosomal proteins directly interact with the signaling receptors of target cells, and exosomes fuse with the plasma membranes of recipient cells to deliver their contents, such as microRNAs, into the cytosol; this process is associated with the pathogenesis of various diseases [2]. Exosomes have a protein and lipid composition that depends on their cellular origin and the state of activation, infection, and/or transformation of the parent cells. These proteins and lipids play roles in mediating inflammatory and autoimmune diseases. For example, vesicles derived from synovial fibroblasts of patients with rheumatoid arthritis (RA) have higher levels of a membrane-bound form of tumor necrosis factor (TNF) than vesicles from healthy control individuals [3]. Lee et al. reported that circulating exosomes from patients with systemic lupus erythematosus (SLE) could induce healthy peripheral blood mononuclear cells to produce inflammatory cytokines, and exosome levels were correlated with disease activity in patients with SLE [4].

RA is a chronic autoimmune disease that is characterized by severe tissue damage and chronic synovial inflammation, eventually leading to joint destruction and disability [5]. Lymphatic vessel endothelial hyaluronic acid receptor-1 (LYVE-1) is known as a marker for lymphangiogenesis, which is also a considerable process for disease progression and potentially exacerbates inflammatory cell persistence. It is reported that LYVE-1+ macrophages were present in RA synovium [6].

Levels of systemic acute-phase reactants (APRs), such as C-reactive protein (CRP), are known to be increased during active synovitis [7]. Serum APR is commonly measured as an indicator of disease severity, progression, and prognosis in RA. The Disease Activity Score in 28 joints (DAS28) is a measure of general health or global disease and is also an inflammatory marker. Although the DAS28 based on erythrocyte sedimentation rate (DAS28-ESR) and CRP (DAS28-CRP) have been developed and validated as markers, there are also considerable factors such as age and sex in patients with RA [8].

Although methotrexate (MTX) and novel biologics have been developed as “breakthrough” RA therapies, considerable numbers of patients respond insufficiently to the therapy, and it remains problematic. The pathogenesis of RA is so complex and patients show such high heterogeneity in disease manifestations and severity that approximately 30–40% of patients do not have a good response to an optimal dosing regimen of MTX. Better markers for prediction of disease prognosis and for reflection of disease activity should be developed to improve patients’ quality of life. Using analysis of genetic variants, biochemical assays, and proteomic approaches, several promising biomarkers for treatment response have been proposed, including red blood cell MTX polyglutamate levels, single-nucleotide polymorphisms, and other genetic variants, as well as serum levels of proteins such as cytokines, growth factors, and autoantibodies [9]. However, these markers need further development and refinement to attain sufficient sensitivity and specificity.

In this study, we used a proteomic approach to identify new exosomal protein biomarkers that are related to disease activity in patients with RA who showed inadequate response to treatment. We also examined the relationship between the levels of these proteins and various serologic parameters of the patients.

## Methods

### Study population and sample preparation

We recruited 60 random female patients with RA who had received MTX for at least 6 months before blood sample collection at Eulji University Hospital in Korea between January 2015 and August 2015. All of the patients met the 2010 American College of Rheumatology/European League Against Rheumatism RA classification criteria [10]. Disease activity was measured using DAS28-ESR. Patients with RA were stratified according to the following criteria: the clinical remission (CR) group (*n* = 30) with a DAS28-ESR ≤2.6 and the non-CR group with a DAS28-ESR >2.6.

For purification and precipitation of serum extracellular vesicles, ExoQuick solution (System Biosciences, Inc., Palo Alto, CA, USA) was used according to the manufacturer’s instructions. Briefly, the serum sample was centrifuged at 3000 × *g* for 15 minutes to remove cells and cell debris. After centrifugation, 250 μl of the serum sample was mixed with 63 μl of ExoQuick solution and incubated for 30 minutes at 4 °C followed by a second centrifugation step at 1500 × *g* for 30 minutes. The supernatant was discarded, and the tubes were centrifuged once more (1500 × *g* for 5 minutes). All traces of fluid were aspirated, and then the tubes were refilled to the starting volume (250 μl) with distilled water. Total exosomal proteins were extracted as described in the Total Exosome RNA and Protein isolation kit (Life Technologies, Carlsbad, CA, USA). For liquid chromatography-tandem mass spectrometry (LC-MS/MS) study, we used the same amounts pooled protein (900 μg) with equal amounts of protein from individuals (30 μg) in each group. Pierce Top 12 Abundant Protein Depletion Spin Columns (Thermo Fisher Scientific, Waltham, MA, USA) were used for removing excess proteins from the serum.

### Tandem mass tag labeling

Samples were subsequently labeled with tandem mass tags (TMTs) for quantitative mass spectrometry (TMTsixplex Isobaric Mass Tagging Kit; Thermo Fisher Scientific). Briefly, each sample was reduced with 500 mM tris(2-carboxyethyl)phosphine at 55 °C for 1 h and then alkylated with 300 mM iodoacetamide at 37 °C in the dark for 30 minutes. The samples were desalted using a 10,000 Da molecular weight cutoff membrane filter and dissolved in 100 mM triethylammonium bicarbonate (TEAB) buffer to a final concentration of 1 μg/μl. Sequencing-grade trypsin (Promega, Madison, WI, USA) was added at 1:100 (wt/wt) to the proteins in TEAB buffer and incubated overnight at 37 °C. Three samples each from the CR and non-CR groups were individually labeled using TMT-126, TMT-128, and TMT-130 (CR group) and TMT-127, TMT-129, and TMT-131 (non-CR group) following the manufacturer’s instructions. Aqueous hydroxylamine solution (5% wt/vol) was added to quench the reaction. The six samples were then combined, dried by speed-vacuum method, and then dissolved in 50 μl of water containing 0.1% formic acid for LC-MS/MS analysis.

### 2D-LC-MS/MS

The TMT-labeled samples were analyzed using a 2D-LC-MS/MS system consisting of a nanoACQUITY ultraperformance liquid chromatography (LC) System (Waters, Milford, MA, USA) and an Orbitrap Elite linear trap quadrupole (LTQ) mass spectrometer (Thermo Fisher Scientific) equipped with a nanoelectrospray source. A detailed description of 2D-LC-MS/MS analysis can be found in the literature [11]. Briefly, a strong cation exchange (5 μm, 3 cm) column was placed immediately before the C_18_ trap column (inner diameter 180 μm, length 20 mm, and particle size 5 μm; Waters). Peptide solutions were loaded in 5-μl aliquots for each run. Peptides were displaced from the strong cation exchange phase to the C_18_ phase by a salt gradient that was introduced through an autosampler loop and then desalted for 10 minutes at a flow rate of 4 μl/minute. Then, the trapped peptides were separated on a 200-mm homemade microcapillary column consisting of C_18_ (particle size 3 μm, Aqua; Phenomenex, Torrance, CA, USA) packed into 100-μm silica tubing with an orifice inner diameter of 5 μm.

An 11-step salt gradient was performed using 3 μl of 0, 25, 50, 100, 250, and 500 mM acetonitrile (ACN; 0.1% formic acid in water) and 4, 5, 9, and an additional 9 μl at 500 mM ACN (0.1% formic acid in 30% ACN). The mobile phases, A and B, were composed of 0% and 100% ACN, respectively, and each contained 0.1% formic acid. The LC gradient began with 5% B for 1 minute and was ramped to 20% B over 5 minutes, to 45% B over 90 minutes, to 95% B over 1 minute, and remained at 95% B over 13 minutes, and it was then ramped to 5% B for another 5 minutes. The column was re-equilibrated with 5% B for 15 minutes before the next run. The voltage applied to produce an electrospray was 2.0 kV. During the chromatographic separation, the Orbitrap Elite LTQ mass spectrometer was operated in a data-dependent mode. The MS data were acquired using the following parameters: four data-dependent collision-induced dissociation high-energy collisional dissociation (CID-HCD) dual MS/MS scans per full scan; CID scans were acquired in LTQ with two-microscan averaging; full scans and HCD scans were acquired in the Orbitrap Elite mass spectrometer at resolutions of 60,000 and 15,000, respectively, with two-microscan averaging, 35% normalized collision energy in CID, and 45% normalized collision energy in HCD; and ±1 Da isolation window. Previously fragmented ions were excluded for 60 seconds. In CID-HCD dual scans, each selected parent ion was first fragmented by CID and then by HCD.

### Protein identification, quantification, and statistical analysis

The resultant MS/MS spectra were analyzed against the latest UniProt human database (version 2.04.2015). Protein identification, quantification, and analysis were performed with the Integrated Proteomics Pipeline IP2 (Integrated Proteomics Applications, San Diego, CA, USA) using ProLuCID (Prolucid Technologies, Mississauga, ON, Canada), DTASelect2, and Census. The rate of decoy hits in the combined forward and reverse database was <1% of the forward hits. ProLuCID [12] was used to identify peptides with the following parameters: a precursor mass error tolerance of 25 ppm and a fragment ion mass error of 600 ppm. The enzyme was specified as trypsin, and three potential missed cleavages were allowed. TMT modification at the N-terminus and lysine residues and carbamidomethylation at cysteine residues were chosen as static modifications. Oxidation at methionine was chosen as the variable modification. The CID and HCD MS/MS spectra from the same precursor ion are often combined by the software to allow better peptide identification and quantification [13]. We used in-house software in which reporter ions from the HCD spectrum were inserted into the CID spectrum using the same precursor ion as in the previous scan. Reporter ions were extracted from small windows (±20 ppm) around their expected mass-to-charge ratio in the HCD spectrum. The output data files were filtered and sorted to compose the protein list using DTASelect [14] with two or more peptide assignments for protein identification. Quantitative analysis was conducted using Census [15], and the intensity at a reporter ion channel for a protein was calculated as the average of this reporter ion’s intensities from all constituent peptides from the identified protein. The resulting ratios were logarithmically transformed (base = 2) to achieve a normal distribution. The median and SD were calculated, and ratio values were corrected for the median to account for variability among different pairs [16]. Ratios were averaged, and proteins with ratio values beyond *p* < 0.01 in normal distribution were defined as significantly regulated. To further assess the individual statistical significance of the expression level change in each protein, one-sample *t* tests were used.

### Measurement of human amyloid A and lymphatic vessel endothelial hyaluronic acid receptor-1 levels by enzyme-linked immunosorbent assay

Human serum and exosome AA concentrations were measured using a commercially available enzyme-linked immunosorbent assay (ELISA) kit (MyBiosource, San Diego, CA, USA). Human serum or exosome samples (300 μg or 30 μg of protein) and the human serum AA calibrator (100 μl) were added to wells coated with antihuman serum AA and incubated at room temperature for 60 minutes. After washing the plate, 100 μl of an enzyme antibody conjugate was added to each well and incubated in the dark at room temperature for 20 minutes. After washing the plate, 100 μl of 3,3′,5,5′-tetramethylbenzidine substrate solution was added to each well and incubated in the dark at room temperature for precisely 10 minutes. After 10 minutes, 100 μl of stop solution was added to each well, and the absorbance was measured on an ELISA reader (xMark Microplate Absorbance Spectrophotometer; Bio-Rad Laboratories, Hercules, CA, USA) at a wavelength of 450 nm. Human serum and exosomal LYVE-1 levels were measured using an ELISA kit following the manufacturer’s instructions.

### Statistical analysis

The normality of the parameters was assessed using the Shapiro-Wilk test. Summary statistics are presented as the mean and SD or as the median and IQR for continuous variables. Comparisons between groups were made using Student’s *t* test or the Mann-Whitney *U* test, as appropriate. *p* Values <0.05 were considered significant. Spearman’s correlation coefficients (ρ) were calculated. The analysis was performed using IBM SPSS Statistics for Windows version 19.0 software (IBM, Armonk, NY, USA).

### Ethics statement

Informed consent was obtained from all of the patients according to the principles of the Declaration of Helsinki.

## Results

### Demographic characteristics and clinical features

The clinical and serological features of the 30 participants in the CR group and the 30 participants in the non-CR group are shown in Table 1. The mean ages of patients were 55.3 years in the CR group and 52.8 years in the non-CR group. There was no significant difference between patients in the two groups with regard to disease duration, rheumatoid factor, or dosage of MTX. Twenty-seven patients (93.1%) in the non-CR group were anti-cyclic citrullinated peptide (anti-CCP) antibody-positive, and 18 patients (64.3%) in the CR group were anti-CCP antibody-positive. The median CRP in the non-CR group was higher than that in the CR group (1.02 vs. 0.09 mg/dl). Four patients (13.3%) in the CR group received oral corticosteroids, compared with 29 patients (96.7%) in the non-CR group.

**Table 1:** Characteristics of patients

Characteristic	CR group	Non-CR group	*p* Value
Age, years, mean ± SD	55.3 ± 10.5	52.8 ± 10.0	0.34
Female sex, *n* (% of total)	30 (100)	30 (100)	N/A
Disease duration, years, median (IQR)	6.1 (4–10.7)	12.35 (3.8–15)	0.26
RF-positive, *n* (% of total)	21 (72.3%)	22 (73.3%)	0.93
Anti-CCP antibody-positive, *n* (% of total)	18 (64.3%)	27 (93.1%)	0.008
CRP, mg/dl, median (IQR)dl	0.09 (0.05–0.14)	1.02 (0.34–2.33)	<0.001
MTX dosage, mg/week, median (IQR)	15 (12.5–15)	15 (15–15)	0.50
Use of corticosteroids, *n* (% of total)	4 (13.3%)	29 (96.7%)	<0.001

*Abbreviations: CCP* Cyclic citrullinated peptide, *CR* Clinical remission, *CRP* C-reactive protein, *MTX* Methotrexate, *RF* Rheumatoid factor

### Proteomic analysis results

After data processing for relative quantification of exosome proteins between the CR and non-CR groups, we identified six candidate proteins (Table 2). AA, fibrinogen β-chain, haptoglobin, and CRP showed differential relative abundance between the two groups, with a fold change >2.83. In particular, AA was more abundant in the non-CR group (fold change 9.06), and LYVE-1 was more abundant in the CR group (fold change 2.23).

**Table 2:** Relative quantification of proteins

Description	Ratio (log_2_ CR/non-CR)
Serum amyloid A1 protein	–3.18
Fibrinogen β-chain	–3.15
Serum amyloid A2 protein	–3.13
C-reactive protein	–2.72
Haptoglobin	–1.51
Lymphatic vessel endothelial hyaluronic acid receptor-1	1.16

*CR* Clinical remission

### Both exosomal and serum AA were more abundant in the non-CR group than in the CR group

**Fig. 1:**
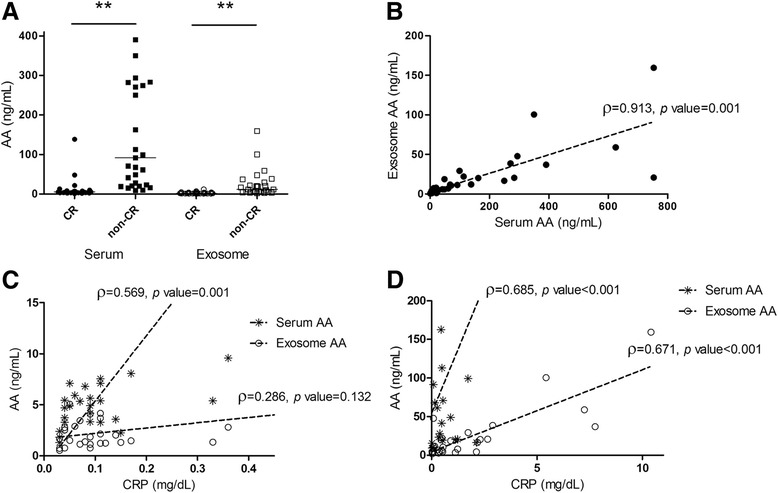
Concentration and correlation of AA. **a** Level of serum AA and exosomal AA in CR group and non-CR group. ** *p* < 0.01. **b** Correlation between exosomal AA and serum AA. **c** Correlation between CRP and AA in CR group (serum and exosome). **d** Correlation between CRP and AA (serum and exosome) in non-CR group. *AA* Amyloid A, *CR* Clinical remission, *CRP* C-reactive protein

To validate these results, we analyzed the level of AA in both groups by ELISA. The non-CR group showed higher levels of both serum and exosomal AA than the CR group; serum AA was 91.5 ng/ml (IQR 22.0–282.9) in the non-CR group and 5.3 ng/ml (IQR 3.52–7.22) in the CR group (*p* = 0.001), and exosomal AA was 16.5 ng/ml (IQR 4.74–40.88) in the non-CR group and 1.9 ng/ml (IQR 1.23–3.51) in the CR group (*p* = 0.001), as shown in Fig. 1a.

We investigated the relationship between serum AA and exosome AA. Serum and exosomal levels of AA were correlated (ρ = 0.913, *p* = 0.001) (Fig. 1b). Serum CRP was significantly correlated with serum AA levels in the CR group (ρ = 0.569, *p* = 0.001) and the non-CR group (ρ = 0.685, *p* < 0.001). Serum CRP was also significantly correlated with exosomal AA in the non-CR group (ρ = 0.671, *p* < 0.001), even though it was not correlated with exosome AA in the CR group (ρ = 0.286, *p* = 0.132) (Fig. 1c and d).

### Level of exosome LYVE-1 was significantly lower in the non-CR group than in the CR group

**Fig. 2:**
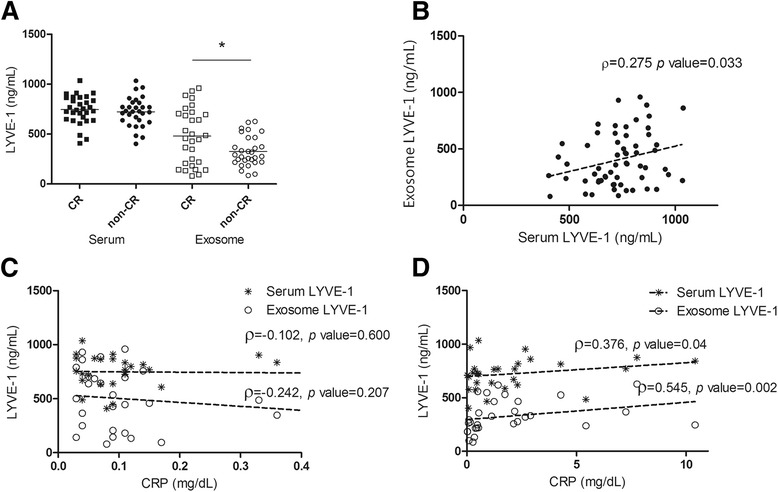
Concentration and correlation of LYVE-1. **a** Level of serum LYVE-1 and exosomal LYVE-1 in CR group and non-CR group. * *p* < 0.05. **b** Correlation between exosomal LYVE-1 and serum LYVE-1. **c** Correlation between CRP and LYVE-1 (serum and exosome) in CR group. **d** Correlation between CRP and LYVE-1 (serum and exosome) in non-CR group. *CR* Clinical remission, *CRP* C-reactive protein, *LYVE-1* Lymphatic vessel endothelial hyaluronic acid receptor 1

We next analyzed the levels of serum LYVE-1 and exosomal LYVE-1 in the CR and non-CR groups by ELISA. The non-CR group showed significantly lower levels of exosomal LYVE-1; exosomal LYVE-1 was 285.0 ng/ml (IQR 219.2–464.8) in the non-CR group and 472.6 ng/ml (IQR 200.3–708.7) in the CR group (*p* = 0.01). However, there was no difference between serum LYVE-1 levels in the CR and non-CR groups: Serum LYVE-1 was 727.0 ng/ml (IQR 633.9–814.2) in the non-CR group and 749.7 ng/ml (IQR 647.4–866.1) in the CR group, as shown in Fig. 2a. Serum and exosomal LYVE-1 were weakly correlated (ρ = 0.275, *p* = 0.033) (Fig. 2b). Moreover, subgroup analysis showed significant correlations between serum CRP and serum/exosomal LYVE-1 (serum ρ = 0.376, *p* = 0.04; exosome ρ = 0.545, *p* = 0.002) in the non-CR group only. In the CR group, there was no significant correlation between serum CRP and serum/exosomal LYVE-1 (serum ρ = −0.102, *p* = 0.600; exosome ρ = −0.242, *p* = 0.207) (Fig. 2c and d).

### Anti-CCP antibody and exosomal LYVE-1 correlation in the non-CR group

Anti-CCP antibody level was significantly negatively correlated with the level of exosomal LYVE-1 in the non-CR group (ρ = −0.453, *p* = 0.014), although no correlation between anti-CCP antibody and exosomal LYVE-1 was observed in the CR group (ρ = 0.043, *p* = 0.828). The correlations between anti-CCP antibody and serum LYVE-1 were not observed in either the CR group (ρ = −0.141, *p* = 0.47) or the non-CR group (ρ = −0.108, *p* = 0.577).

## Discussion

Although several methods have been developed to predict disease activity in patients with RA, their efficacy is insufficient. Researchers in recent studies have used exosome analysis to investigate the intracellular state. We investigated the relationship between disease activity and concentration of exosomal proteins using serum exosomes collected from patients with RA with varying levels of disease activity and employing comparative quantitative proteomic methods. We identified six exosomal proteins that showed significant differences between the two disease activity groups. Both serum and exosomal concentrations of AA tended to be higher in patients with higher disease activity. In contrast, the concentration of LYVE-1 was lower in patients with higher disease activity, especially in exosomes.

Serum AA is linked to the pathogenesis of various diseases, such as atherosclerosis, diabetes, Alzheimer’s disease, and RA [17]. Serum AA reaches much higher levels than CRP and declines rapidly. Growing evidence suggests that serum AA is related to diagnosis, disease activity, and assessment of treatment response in RA. Therefore, serum AA can be useful in predicting the clinical response in relation to disease activity [17, 18]. However, several questions remain unanswered, including which inflammatory conditions increase serum AA and which technologies may be useful for rapid and accurate screening. In our study, both exosomal and serum AA were shown to be associated with RA disease activity, and they may also be useful markers for other chronic inflammatory or autoimmune diseases.

Serum AA is produced in response to proinflammatory cytokines and messenger RNA of AA was found to be upregulated by TNF-α) and interleukin-1 (IL-1), as well as by IL-6 to a lesser extent, in fibroblast-like synoviocytes (FLSs) of patients with RA [19, 20]. However, previous studies suggested that glucocorticoids could induce serum AA levels that could be secreted by chondrocytes and FLSs, mostly under glucocorticoid treatment [21]. Our data show that serum and exosomal AA were increased in the non-CR group, and they also show that the number of patients with glucocorticoid use in the non-CR group was high. However, we did not find a correlation between AA and glucocorticoid use. Further prospective studies are necessary to provide an accurate answer.

LYVE-1 is a hyaluronic acid receptor that is selectively expressed in the endothelium of lymphatic capillaries. Immunohistochemical intensity of LYVE-1 expression is useful for detecting lymphatic invasion or lymphangiogenesis [22]. A recent study showed that LYVE-1 expressed on the lymphatic endothelium may be shed and subsequently released into the circulation, as well as that low levels of LYVE-1 are observed in larger lung cancer tumors with greater metastasis [23]. However, the degradation pathway of serum LYVE-1 has not been investigated. Expression of LYVE-1 is known to be low in inflammatory conditions. Lymphangiogenesis in chronic inflammation has a mechanism different from that in acute inflammation [24]. Elevated expression of LYVE-1 may have a profound effect on lymphangiogenesis, but the major factors triggering LYVE-1 activation under these conditions remain unclear. In this study, we found that exosomal LYVE-1 was lower in the non-CR group than in the CR group, but the level of exosomal LYVE-1 was not correlated with serum CRP or AA. Few markers with negative correlation have been reported in RA, but exosomal LYVE-1 could be used as an additive marker, with further investigation of the pathogenesis and the mechanism of lymphangiogenesis in RA and autoimmune disease.

Biomarkers can help to improve diagnosis and prognosis, as well as assessment of response to treatment, and progress should continue to be made in discovery and development of markers for RA. Changes in levels of specific inflammatory mediators during treatment have been assessed as predictors of treatment response [25, 26]. Treatment with MTX alters the protein profiles of exosomes, and MTX treatment suppresses some of the changes induced by IL-1β in vitro [27]. The protein and microRNA contents of exosomes have been suggested to reflect the physiological conditions of their cells of origin; exosomes also induce phenotypic changes in their origin cells in vitro [28]. These data support the hypothesis that intracellular changes following treatment might alter exosomal contents, such as proteins and RNA. Therefore, exosomes could be a useful tool to assess the response to treatment and to identify specific markers for diagnosis and predict prognosis.

There are several limitations to our study. First, it was limited by the relatively small sample size and the lack of data from healthy control individuals. However, because the protein levels of AA or LYVE-1 differed so greatly between the CR and non-CR groups, we believe our results are still relevant. Second, our study was designed as a cross-sectional study, and further research into the changes in protein concentration should be performed in the future. Third, the study population was selected from among the patients of a university hospital in South Korea and therefore contains an ethnicity selection bias.

## Conclusions

Exosomal AA and exosomal LYVE-1 show promise as additional markers for evaluating disease activity in patients with RA. Prospective investigation of exosomal levels of AA and LYVE-1 may facilitate development of new diagnostic tools to assess disease activity and prognosis in RA and other autoimmune diseases.

## Abbreviations

AA: Amyloid A
Ab: Antibody
ACN: Acetonitrile
APR: Acute-phase reactant
CCP: Cyclic citrullinated peptide
CID: Collision-induced dissociation
CID-HCD: Collision-induced dissociation high-energy collisional dissociation
CR: Clinical remission
CRP: C-reactive protein
DAS28: Disease Activity Score in 28 joints
ELISA: Enzyme-linked immunosorbent assay
ESR: Erythrocyte sedimentation rate
FLS: Fibroblast-like synoviocyte
HCD: High-energy collisional dissociation
IL: Interleukin
LC: Liquid chromatography
LC-MS/MS: Liquid chromatography-tandem mass spectrometry
LTQ: Linear trap quadrupole
LYVE-1: Lymphatic vessel endothelial hyaluronic acid receptor-1
MTX: Methotrexate
RA: Rheumatoid arthritis
RF: Rheumatoid factor
SLE: Systemic lupus erythematosus
TEAB: Triethylammonium bicarbonate
TMT: Tandem mass tag
TNF: tumor necrosis factor
